# Tumor Necrosis Factor-Alpha (+489 G/A) Polymorphism Can Predict the Response to Adalimumab in Chinese Han Patients With Ankylosing Spondylitis

**DOI:** 10.7759/cureus.42704

**Published:** 2023-07-30

**Authors:** Hong-Jin Liang, Xiao-Min Jiang, Feng-Cai Shen, Jian-Hua Peng, Dan-Min Wang, Shu-Xin Huang, Zhi-duo Hou, Ling Lin

**Affiliations:** 1 Department of Rheumatology and Immunology, The First Affiliated Hospital of Shantou University Medical College, Shantou, CHN; 2 Clinical Research Center, The First Affiliated Hospital of Shantou University Medical College, Shantou, CHN

**Keywords:** ankylosing spondylitis, single nucleotide polymorphism, adalimumab, tumor necrosis factor-alpha, tumour necrosis factor-α (tnf-α), biological therapy, adalimumab (humira)

## Abstract

Background: Studies investigating the association between single nucleotide polymorphisms (SNPs) of tumor necrosis factor-alpha (TNFα) and the efficacy of adalimumab (ADA) in ankylosing spondylitis (AS) therapy have reported conflicting results. We aimed to investigate the value of SNP typing of *TNFα* in predicting the efficacy of ADA in AS.

Materials and methods:Eighty patients with active AS who received ADA treatment were followed up for 24 weeks. Six known SNPs of *TNFα* (+489G/A, -238G/A, -308G/A, -857C/T, -863C/A, and -1031C/T) were subjected to the SNaPshot SNP typing method, which has been proven to be a reliable, efficient, and cost-effective method for detecting SNPs. The relationship between each SNP genotype and the therapeutic efficacy of ADA was analyzed.

Results: At the end of the 24-week follow-up, 58.8% of the patients with AS achieved Assessment of SpondyloArthritis International Society (ASAS) partial remission (PR), 67.5% of the patients achieved the criteria of an ASAS40 response (40% improvement on indices), and 53.8% of the patients achieved Ankylosing Spondylitis Disease Activity Score (ASDAS) major improvement (MI). The univariate analysis showed that patients with AS carrying the *TNFα *+489 A allele were more likely to achieve ASAS-PR, ASAS40 response criteria, and ASDAS-MI after ADA treatment. In the multivariate regression analysis, the *TNFα *+489 A allele was an independent factor influencing the efficacy of ADA in treating AS (ASAS-PR odds ratio (OR) = 2.66, 95% confidence interval (CI) = 1.01-7.01; ASAS40 OR = 4.56, 95% CI = 1.39-15.00; ASDAS-MI OR = 3.31, 95% CI = 1.02-10.69).

Conclusions: The patients carrying the *TNFα* +489 A allele may be more likely to experience better therapeutic efficacy and achieve the treatment target (ASAS-PR, ASAS40 response, or ASDAS-MI) after receiving ADA treatment. Detection of *TNFα* +489 G/A may predict the therapeutic efficacy of ADA, which can be used in clinical practice to tailor treatment for individual patients with AS. Further studies with larger sample sizes and longer follow-up periods with imaging evaluation are needed to verify our findings.

## Introduction

Ankylosing spondylitis (AS) is an inflammatory disease that mainly involves the axial joints, and it may be accompanied by peripheral joint involvement and extra-articular manifestations [1]. Most patients with AS in China have an insidious disease onset, and the average course of the disease at the time of diagnosis is more than six years [2]. The development of ankylosis and spinal deformity in the late stage of the disease can lead to disability and unemployment, which not only seriously affects the economic status and quality of life of patients but also imposes a heavy burden on their families and society. Therefore, early treatment is the key to a good prognosis. In recent years, adalimumab (ADA), a fully human monoclonal antibody against tumor necrosis factor-alpha (TNFα), has achieved good results in AS treatment [3], which has improved patients’ quality of life. The results of a phase III clinical trial in Chinese adults with active AS showed that ADA treatment resulted in a significantly higher percentage of Assessment of SpondyloArthritis International Society (ASAS) 20 (20% improvement in indices) response than placebo [4], suggesting that ADA has a good effect in most patients with AS in China. However, nearly one-third of patients with AS still show a poor response to ADA, and the drug is expensive. Therefore, effective prediction of response to ADA treatment in Chinese AS patients is conducive to achieving better individualized and precise treatment and effectively saving treatment costs.

There are several factors affecting individuals’ therapeutic response to drugs, such as heredity, environment, and disease state. Among them, genetic factors are particularly important. Previous research has shown that single nucleotide polymorphisms (SNPs) of *TNFα* may affect the expression of TNFα and consequently the therapeutic efficacy of TNFα inhibitors (TNFi) [5]. However, the related conclusions vary among studies. A meta-analysis suggested that these differences among studies may be related to race, methods for evaluating therapeutic efficacy, and TNFi types. To date, pharmacogenomics research on ADA has mainly focused on psoriatic arthritis (PsA) and inflammatory bowel disease, and there is a lack of systematic research on AS. In this study, we aimed to analyze the relationship between six reported SNPs of *TNFα* and the therapeutic efficacy of ADA and investigate the feasibility of predicting the therapeutic efficacy of ADA in Chinese patients with AS by SNP typing of *TNFα*.

## Materials and methods

Study design and participants

This single-center, prospective cohort study was conducted in the First Affiliated Hospital of Shantou University Medical College between July 2017 and June 2021. Eighty eligible patients with AS were enrolled, and all patients fulfilled the modified New York Criteria for AS [6], who presented with an inadequate response or were intolerant to ≥2 non-steroidal anti-inflammatory drugs (NSAIDs) and had active disease (as defined by at least one of the following: Bath Ankylosing Spondylitis Disease Activity Index (BASDAI) ≥ 4.0 and Ankylosing Spondylitis Disease Activity Score (ASDAS)-C-reactive protein (CRP) ≥ 2.1). Patients with acute or chronic infections (including tuberculosis), severe cardiac, hepatic, and renal insufficiency, nervous system diseases, and tumors were excluded. The patients were treated with ADA (40 mg, subcutaneous injection, every two weeks) and followed up for 24 weeks. Concomitant use of sulfasalazine (≤3 g/day) and NSAIDs was allowed, but the dose had to be stable for more than two weeks for NSAIDs and for more than four weeks for sulfasalazine at the time of screening. The study was approved by the Ethics Committee of the First Affiliated Hospital of Shantou University Medical College (B-2017-045), and informed consent was obtained from all participants.

Clinical data and main outcomes

The general demographic data, baseline clinical characteristics, and various evaluation indices (BASDAI, Bath Ankylosing Spondylitis Functional Index (BASFI), ASDAS-CRP, and ASDAS-erythrocyte sedimentation rate (ESR)) were recorded. Efficacy assessments were performed at weeks zero, four, 12, and 24. The primary efficacy assessment was the percentage of patients achieving ASAS partial remission (ASAS-PR) at week 24, as defined in a previous report [7]. Additional endpoints assessed at week 24 were the percentage of patients achieving the following outcome measures: ASAS40 response criteria [7] and ASDAS major improvement (ASDAS-MI) [8].

SNP typing

Genomic DNA was extracted from peripheral blood samples using the QIAamp Blood Kit (QIAGEN, Hilden, Germany) and stored at −80°C. Genotyping of six *TNFα* SNPs (+489G/A, -238G/A, -308G/A, -857C/T, -863C/A, and -1031C/T) was performed using the SNaPshot technique with the following primers: -857C/T, -863C/A, and -1031C/T: F-GGAGAACAAAAGGATAAGGGCTCA, R-GTCCTGGAGGCTCTTTCACTCC; +489G/A: F-CTCTTCTGCCTGCTGCACTTTG, R-CCCCATCTCTTGCCACATCTCT; -308G/A and -238G/A: F-CTTTCTGAAGCCCCTCCCAGTT, R-AGTTGGGGACACACAAGCATCA.

Statistical analysis

IBM SPSS Statistics for Windows (released 2019, version 26.0., IBM Corp., Armonk, NY) was used for statistical analysis. The Hardy-Weinberg equilibrium test was used to analyze the group representativeness of case samples. Measurement data conforming to a normal distribution or close to a normal distribution are expressed as mean ± standard deviation, and comparisons between groups were made using independent samples t-test. Measurement data not conforming to a normal distribution are expressed as median (interquartile range (IQR)), and comparisons between groups were performed using the Mann-Whitney U rank-sum test. The chi-square test or Fisher’s exact test was used for enumeration data. Odds ratio (OR) and 95% confidence interval (CI) were calculated using multivariable logistic models to identify the variables (SNP, fatigue, spinal pain, peripheral arthritis, BASDAI, ASDAS-CRP, and ASDAS-ESR) that contributed to the variability of the efficacy of ADA treatment and covariate analysis was used to adjust for confounding variables. P < 0.05 was considered statistically significant.

## Results

Therapeutic efficacy of ADA for 24 weeks in patients with AS

**Figure 1:**
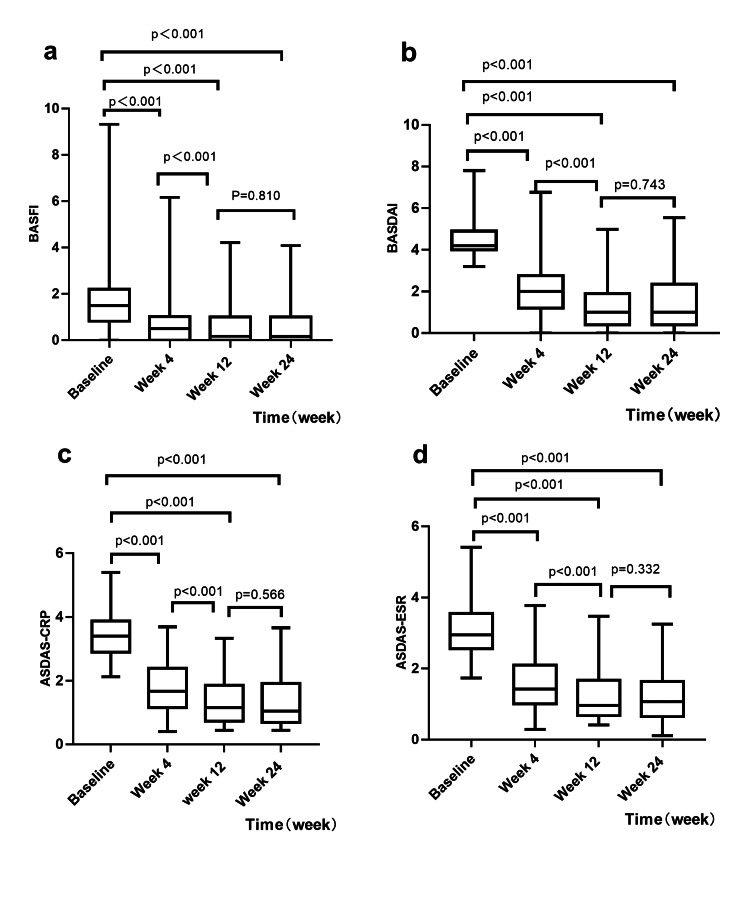
Changes in disease activity scores during the 24-week follow-up Changes were based on (a) Bath Ankylosing Spondylitis Functional Index (BASFI), (b) Bath Ankylosing Spondylitis Disease Activity Index (BASDAI), (c) Ankylosing Spondylitis Disease Activity Score-C-reactive protein (ASDAS-CRP), and (d) Ankylosing Spondylitis Disease Activity Score-erythrocyte sedimentation rate (ASDAS-ESR).

Eighty patients with AS (61 men and 19 women) treated with ADA were enrolled; their median age was 26.0 (10.0) years and the median course of the disease was 6.0 years. All patients were followed up for 24 weeks according to the study design. The proportion of ADA-administered patients who achieved ASAS-PR, ASAS40 response, and ASDAS-MI at week four was 31.3% (25/80), 42.5% (34/80), and 33.8% (27/80), respectively. Compared with baseline values, the ASDAS-CRP, ASDAS-ESR, BASFI, and BASDAI were significantly improved in patients with AS (Figure 1).

At week 12, 55.0% (44/80) of the patients achieved ASAS-PR, 63.8% (51/80) of the patients achieved an ASAS40 response, and 55.0% (44/80) of the patients achieved ASDAS-MI. Compared with those at baseline and four weeks, the ASDAS-CRP, ASDAS-ESR, BASFI, and BASDAI were significantly improved in patients with AS (Figure 1).

At the endpoint, the proportion of ADA-administered patients who achieved ASAS-PR, ASAS40 response, and ASDAS-MI was 58.8% (47/80), 67.5% (54/80), and 53.8% (43/80), respectively. Compared with those at baseline and four weeks, the mean ASDAS-CRP, ASDAS-ESR, BASFI, and BASDAI were significantly improved, but there was no significant difference in any of the measurements compared with those at week 12 (P > 0.05) (Figure 1).

Relationship between SNPs of TNFα and efficacy of ADA in AS treatment

The distribution of genotypes for all SNPs conformed to the Hardy-Weinberg equilibrium (data not shown). The mutation frequency of *TNFα* -238G/A was too low; therefore, this SNP was not included in the analysis of the relationship with the therapeutic efficacy of ADA. The relationships between the remaining five SNPs (+489G/A, -308G/A, -857C/T, -863C/A, and -1031C/T) and the therapeutic efficacy of ADA are summarized in Table 1. In the univariate analysis, there were significant differences in the proportion of patients who achieved ASAS-PR, ASAS40 response, and ASDAS-MI at the endpoint among different genotypes of *TNFα* +489. The proportion of patients with AS who achieved ASAS-PR, ASAS40 response, and ASDAS-MI was higher in those carrying *TNFα* +489 genotypes GA and AA (A allele) than in those carrying genotype GG (ASAS-PR: 72.3% vs. 27.7%, P = 0.030; ASAS40: 72.2% vs. 27.8%, *P* = 0.10; ASDAS-MI: 74.4% vs. 25.6%, *P *= 0.018). The genotype frequencies of the other four SNPs were not significantly associated with any of the three efficacy indices at the study endpoint.

**Table 1:** Influencing factors of efficacy of adalimumab in patients with ankylosing spondylitis * Data are expressed as median (IQR), and comparisons between two groups were performed with the Mann-Whitney U rank-sum test. # Data are expressed as mean ± standard deviation, and comparisons between two groups were made by independent-samples t-test. § Fisher’s exact test was used. TNFα: tumor necrosis factor-alpha; NS: no statistical significance in univariate analysis; NI: the difference was statistically significant in univariate analysis, but was not included in the multivariate regression analysis; HLA-B27: human leukocyte antigen B27; PGA: patient global assessment; VAS: visual analog scale; BASFI: Bath Ankylosing Spondylitis Functional Index; BASDAI: Bath Ankylosing Spondylitis Disease Activity Index; ASDAS: Ankylosing Spondylitis Disease Activity Score; CRP: C-reactive protein; ESR: erythrocyte sedimentation rate.

Risk factor	ASAS-PR responders		Multivariate analysis			ASAS40 responders		Multivariate analysis			ASDAS-MI responders		Multivariate analysis	
	Yes (n = 47)	No (n = 33)	P-value	OR (95% CI)		Yes (n = 54)	No (n = 26)	P-value	OR (95% CI)		Yes (n = 43)	No (n = 37)	P-value	OR (95% CI)
Male, n (%)	37 (78.7)	24 (72.7)		NS		43 (79.6)	18 (69.2)		NS		36 (83.7)	25 (67.6)		NS
Disease onset^#^, years	20.1 ± 6.1	21.3 ± 5.6		NS		20.4 ± 5.8	21.2 ± 6.1		NS		19.6 ± 5.8	21.8 ± 5.8		NS
Disease duration*, years	6.0 (5.0)	6.0 (7.0)		NS		6.5 (6.0)	6.0 (6.3)		NS		6.0 (6.0)	6.0 (7.0)		NS
HLA-B27 (+), n (%)	43 (91.5)	25 (75.8)		NS^§^		49 (90.7)	19 (73.1)		NS^§^		38 (88.4)	30 (81.1)		NS
PGA (VAS)*	6.7 (1.8)	7.0 (2.0)		NS		6.8 (1.0)	7.0 (1.2)		NS		7.0 (1.0)	6.0 (2.0)		NS
Spine pain (VAS)*	6.0 (2.0)	6.0 (2.0)		NS		6.0 (2.0)	5.6 (2.7)		NS		6.0 (2.0)	6.0 (1.0)		NI
Peripheral arthritis (VAS)*	3.0 (5.0)	4.0 (5.5)		NS		3.0 (5.0)	4.5 (4.0)	0.001	0.59 (0.44-0.80)		4.0 (4.4)	3.0 (5.0)		NS
Fatigue (VAS)^ #^	4.1 ± 1.9	3.1 ± 2.2	0.03	1.30 (1.02-1.65)		4.1 ± 2.0	2.8 ± 2.0		NI		4.1 ± 2.0	3.2 ± 2.0	0.01	1.58 (1.14-2.19)
BASFI*	1.1 (1.1)	2.0 (2.2)		NS		1.5 (1.2)	1.0 (1.4)		NS		1.5 (1.2)	1.0 (1.4)		NS
BASDAI*	4.2 (0.9)	4.1 (0.6)		NS		4.2 (1.1)	4.0 (0.5)	0.001	5.23 (2.06-13.28)		4.4 (1.3)	4.0 (0.4)		NI
ASDAS-CRP^#^	3.3 ± 0.7	3.4 ± 0.8		NS		3.4 ± 0.7	3.2 ± 0.7		NS		3.7 ± 0.7	3.0 ± 0.6	0.001	9.39 (3.05-28.89)
ASDAS-ESR^#^	3.1 ± 0.8	3.1 ± 0.8		NS		3.1 ± 0.8	2.9 ± 0.6		NS		3.4 ± 0.8	2.7 ± 0.6		NI
TNFα –857														
T allele (CT+TT), n (%)	33 (70.2)	19 (57.6)		NS		38 (70.4)	14 (53.8)		NS		31 (72.1)	21 (56.8)		NS
TNFα +489														
A allele (AA+GA), n (%)	34 (72.3)	16 (48.5)	0.048	2.66 (1.01-7.01)		39 (72.2)	11 (42.3)	0.01	4.56 (1.39-15.00)		32 (74.4)	18 (48.6)	0.046	3.31 (1.02-10.69)
TNFα –308														
A allele (GA+AA), n (%)	12 (25.5)	5 (15.2)		NS		12 (22.2)	5 (19.2)		NS		10 (23.3)	7 (18.9)		NS
TNFα –863														
A allele (CA+AA), n (%)	14 (29.8)	6 (18.2)		NS		15 (27.8)	5 (19.2)		NS		13 (30.2)	7 (18.9)		NS
TNFα –1031														
C allele (CC+CT), n (%)	18 (38.3)	7 (21.2)		NS		19 (35.2)	6 (23.1)		NS		15 (34.9)	10 (27.0)		NS

In the multivariate analysis, the clinical characteristics of patients at baseline were included as independent variables in the logistic regression analysis, including numerical rating scale (NRS) of fatigue, spinal pain, and peripheral arthritis, BASDAI, ASDAS-CRP, ASDAS-ESR (which might be related to therapeutic efficacy according to the univariate analysis), and *TNFα* +489 A allele. The final multivariable model analysis adjusted for confounders revealed that regardless of the efficacy assessment index used (ASAS-PR, ASAS40 response, or ASDAS-MI), the *TNFα* +489 A allele was an independent factor associated with the efficacy of ADA in patients with AS (ASAS-PR: OR = 2.66, 95% CI = 1.01-7.01; ASAS40 response: OR = 4.56, 95% CI = 1.39-15.00; ASDAS-MI: OR = 3.31, 95% CI = 1.02-10.69) (Table 1).

## Discussion

International and regional recommendations/guidelines recommend using TNFi in AS patients with persistently high disease activity despite conventional treatment [9-11]. In this study, for the patients who received ADA, clinical indices such as ASDAS-CRP, ASDAS-ESR, BASFI, and BASDAI significantly improved after four weeks of treatment compared with those measured at baseline, which could be further improved after 12 weeks of treatment, although there was no significant difference in any of the clinical indices between 12 and 24 weeks (Figure 1). These findings indicated that the disease activity and functions of the majority of patients with AS were improved after receiving ADA treatment for four weeks, and the plateau of the maximum therapeutic efficacy could be reached after 12 weeks.

In recent years, the concept of “treat to target” has been introduced into AS treatment [12]. As clinical remission is an important parameter of treat to target, ASAS-PR was selected as the primary efficacy assessment for therapeutic efficacy evaluation of ADA in this study. The ASAS response criteria have been widely applied in evaluating the therapeutic efficacy of biological agents for treating AS. However, ASAS response criteria do not include the assessment of acute-phase reactants. Therefore, in this study, we also used ASAS40 response criteria and ASDAS-MI as secondary efficacy assessments, which would ensure a more objective and rigorous evaluation of the therapeutic efficacy of ADA and the disease condition. At the end of the follow-up, most of the patients with AS achieved a good therapeutic response, with more than half of the patients achieving ASAS-PR, ASAS40 response, and ASDAS-MI, suggesting that ADA is effective in Chinese patients with AS.

To further explore the influences of genetic factors on the efficacy of ADA in treating AS, we conducted genotyping for six SNPs of TNFα and analyzed whether SNP genotyping can be used as a predictor of ADA therapeutic efficacy. In the univariate analysis, there were differences in the primary efficacy assessment index (ASAS-PR) and the secondary efficacy assessment indices (ASAS40 response and ASDAS-MI) among different genotypes at *TNFα *+489. *TNFα* +489 is located in the intron region, and the G/A mutation in this site may affect the biological function of *TNFα* to express a higher serum TNFα level [13]. Early research on rheumatoid arthritis (RA) showed that the G/A point mutation at locus *TNFα* +489 could increase the susceptibility to RA, and it was also related to radiographic damage in RA [14]. Moreover, the A allele at *TNFα* +489 has been reported to be related to disease susceptibility, the therapeutic efficacy of ADA, and the progression of joint erosions in PsA [15]. However, to the best of our knowledge, the relationship between this SNP and the therapeutic efficacy of ADA in AS has not been studied before.

In this study, the proportion of AS patients who achieved ASAS-PR, ASAS40 response, and ASDAS-MI was higher in those carrying the *TNFα* +489 A allele (genotypes GA and AA) than in those carrying genotype GG after 24 weeks of ADA treatment. This suggested that patients with AS carrying the *TNFα* +489 A allele may be more likely to achieve the treatment target and experience better therapeutic efficacy after receiving ADA treatment. Considering that the baseline clinical characteristics of patients with AS may also affect the therapeutic efficacy of ADA, to adjust for these confounding factors, we included variables with statistical significance in the univariate analysis (baseline fatigue level, spinal pain level, peripheral arthritis, BASDAI, ASDAS-CRP, and ASDAS-CRP) in the multivariate analysis along with the SNPs. The results showed that regardless of the efficacy assessment index selected (ASAS-PR, ASAS40 response, or ASDAS-MI), the *TNFα* +489 A allele was an independent factor influencing the therapeutic efficacy of ADA in treating AS. The present study demonstrated for the first time an association between the *TNFα* +489 A allele and the ADA good response in AS patients. According to the results of the biological function study of the +489 A allele [13], we speculate that patients carrying this gene have higher levels of tumor necrosis factor-related inflammatory factors, and the disease can be alleviated more quickly after ADA treatment.

Several studies designed to evaluate the association between some *TNFα* gene polymorphisms at positions -308, -857, -863, and -1031, and response to anti-TNFα therapy, have reported conflicting results [16-19]. We did not find significant associations between the *TNFα* SNPs at position -308, -857, -863, and -1031 and the therapeutic efficacy of ADA in Chinese patients with AS, although there was a trend for association with the *TNFα* -857 T allele, which was previously found to be associated with better treatment response to etanercept in patients with RA [20]. This could be attributed to the following reasons. First, AS, RA, and PsA are different diseases and may have different mechanisms for the response to TNFi. Second, different TNFis were used in previous studies. Third, different efficacy assessments were adopted.

Interestingly, we found that the fatigue level at baseline was also a weak influencing factor on the therapeutic efficacy of ADA. Previous clinical research showed that ADA could effectively improve the fatigue of patients with AS [21]. Therefore, we believe that a more severe fatigue level at baseline can result in a more significant improvement after ADA treatment. This study had some limitations. First, the study is conducted at a single center with a relatively small sample size of Chinese Han patients with AS, which may limit the generalizability of the findings to other populations or ethnicities. Second, the 24-week follow-up period may not be sufficient to evaluate the long-term therapeutic response. Third, the lack of imaging evaluation in the spine and sacroiliac joint may have limited the ability to fully assess the efficacy, which could impact the accuracy and completeness of the study's conclusions. In particular, the A allele at locus +489 has been suggested to be related to joint erosions in RA and PsA in previous studies [14,15]. Therefore, the conclusion needs to be further verified in studies with a higher number of patients, involving multiple centers, and a longer course of treatment.

## Conclusions

In summary, although most patients with AS benefit from ADA treatment, more than 40% fail to achieve the treatment target (ASAS-PR) after 24 weeks of ADA treatment. In this study, we found the association of *TNFα* +489 G/A with the response to ADA in Chinese Han patients with AS. The patients carrying the *TNFα* +489 A allele may be more likely to experience better therapeutic efficacy and achieve the treatment target (ASAS-PR, ASAS40 response, or ASDAS-MI) after receiving ADA treatment. Detection of *TNFα* +489 G/A may predict the therapeutic efficacy of ADA, which can be used in clinical practice to tailor treatment for individual patients with AS. Due to the limitation of this study, including a small number of patients and a short follow-up period, further studies with larger sample sizes and longer follow-up periods with imaging evaluation are needed to verify our findings.
